# Protein Inhibitor of Activated STAT, PIASy Regulates α-Smooth Muscle Actin Expression by Interacting with E12 in Mesangial Cells

**DOI:** 10.1371/journal.pone.0041186

**Published:** 2012-07-19

**Authors:** Kazuo Torikoshi, Hideharu Abe, Takeshi Matsubara, Takahiro Hirano, Takayuki Ohshima, Taichi Murakami, Makoto Araki, Akira Mima, Noriyuki Iehara, Atsushi Fukatsu, Toru Kita, Hidenori Arai, Toshio Doi

**Affiliations:** 1 Department of Nephrology, Kyoto University Graduate School of Medicine, Kyoto, Japan; 2 Department of Nephrology, Health-Bioscience Institute, University of Tokushima Graduate School of Medicine, Tokushima, Japan; 3 Faculty of Pharmaceutical Science at Kagawa Campus, Tokushima Bunri University, Kagawa, Japan; 4 Kobe City Medical Center General Hospital, Kobe, Japan; 5 Department of Human Health Sciences, Kyoto University Graduate School of Medicine, Kyoto, Japan; North Carolina State University, United States of America

## Abstract

Phenotypic transformation of mesangial cells (MCs) is implicated in the development of glomerular disease; however, the mechanisms underlying their altered genetic program is still unclear. α-smooth muscle actin (α-SMA) is known to be a crucial marker for phenotypic transformation of MCs. Recently, E-boxes and the class I basic helix-loop-helix proteins, such as E12 have been shown to regulateα-SMA expression. Therefore, we tried to identify a novel E12 binding protein in MCs and to examine its role in glomerulonephritis. We found that PIASy, one of the protein inhibitors of activated STAT family protein, interacted with E12 by yeast two-hybrid screens and coimmunopreciptation assays. Overexpression of E12 significantly enhanced theα-SMA promoter activity, and the increase was blocked by co-transfection of PIASy, but not by a PIASy RING mutant. *In vivo* sumoylation assays revealed that PIASy was a SUMO E3 ligase for E12. Furthermore, transforming growth factor-β (TGF-β) treatment induced expression of both PIASy and E12, consistent with α-SMA expression. Moreover, reduced expression of PIASy protein by siRNA specific for PIASy resulted in increased TGF-β-mediated α-SMA expression. *In vivo*, PIASy and E12 were dramatically upregulated along with α-SMA and TGF-β in the proliferative phase of Thy1 glomerulonephritis. Furthermore, an association between PIASy and E12 proteins was observed at day 6 by IP-western blotting, but not at day 0. These results suggest that TGF-β up-regulates PIASy expression in MCs to down-regulateα-SMA gene transcription by the interaction with E12.

## Introduction

Phenotypic modulation of mesangial cells (MCs) in response to injury is implicated in the development of glomerular disease. Activation of MCs *in vivo* contributes to hyperplasia or hypertrophy and marked accumulation and reorganization of the mesangial matrix, all of which precede glomerulosclerosis [1], [2], [3]. Therefore, it is important to elucidate the transcriptional mechanisms underlying the altered genetic program of MCs and glomerulosclerosis. α-SMA has been shown to be a crucial marker for activation and dedifferentiation of MCs [4], [5]. Although elucidating transcriptional mechanisms regulating α-SMA expression in MCs should yield some insight into genetic mechanisms that determine the activated phenotype, little is known about the regulation of α-SMA expression in MCs.

The 5′-flanking region of the α-SMA gene contains conserved E-boxes (CANNTG motifs). E-boxes bind to homo- or heterodimers of basic helix loop helix (bHLH) proteins, with the general paradigm being heterodimerization between a ubiquitously expressed class I bHLH protein and a cell-selective class II bHLH protein. Kumar et al demonstrated that the two E-boxes found within the 5′ region of the α-SMA promoter were required for the expression in transgenic mice [6]. Furthermore, they provided evidence that the class I bHLH proteins (including E2-2, E12, and HEB) are involved in α-SMA regulation in cultured smooth muscle cells. On the other hand, a number of studies have suggested that E2A proteins related to epithelial mesenchymal transition (EMT) [7], [8]. Accordingly, we postulated that the class 1 bHLH factor E2A protein (E12/E47, especially E12) is also involved in α-SMA regulation in MCs.

The E2A gene encodes two alternatively spliced products, E12 and E47, which differ only in their bHLH domains [9]. Both proteins are involved in the control of cell-specific differentiation and cell proliferation [10], [11]. Regulation of HLH factors has been shown to occur through multiple mechanisms including protein expression, phospholylation, dimerization with other HLH factors, cell localization, ubiquitination, and subsequent proteasome degradation [12], [13]. Therefore, strict control of HLH levels and activity is necessary to prevent uncontrolled cell proliferation and dedifferentiation and may be necessary for tissue repair following injury.

The aims of our study were two folds: (i) to identify novel classes of proteins that interact with E12 and modulate expression of α-SMA in MCs by utilizing yeast two-hybrid screening; and (ii) to clarify the role of these genes in experimental mesangial proliferative glomerulonephritis.

## Results

### Cloning of PIASy as E12-interacting Proteins

To identify novel classes of proteins that interact with E12 and modulate expression of α-SMA in MCs, we performed yeast two-hybrid screening using a mouse mesangial cDNA/GAL4 activation domain fusion library and the region between amino acids 505 and 651 including bHLH of E12 as bait. From 1.5 x 10^6^ independent transformants, three different clones were found to be true interacting positives on selection medium SD/−His/−leu/−Trp plate only when they were co-expressed with Gal4-E12-bHLH fusion protein. DNA sequence analysis of the plasmid insert and computer-assisted database search revealed that the nucleotide sequence of one clone encoded a member of the protein inhibitor of activated STAT (signal transducers and activators of transcription) family, PIASy.

**Figure 1:**
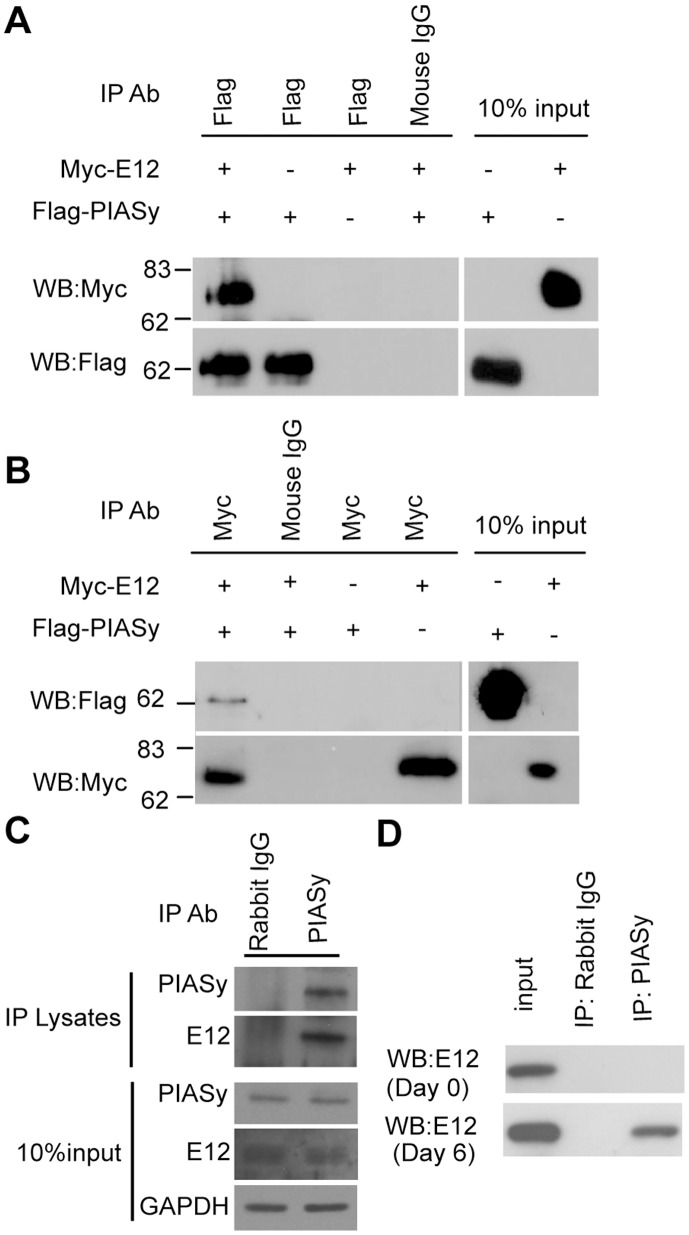
Interaction of E12 with PIASy. (A) Coimmunoprecipitation of E12 and PIASy. COS7 cells were transfected with expression plasmids encoding the indicated proteins and incubated for 48 hours. Cell lysates were then prepared, immunoprecipitated (IP) with anti-flag antibody, and analyzed by Western blotting (WB) with anti-myc (top) or anti-flag (bottom) antibodies (Ab). Mouse IgG was used as a control. Input materials (10%) were used as a reference standard. Molecular size markers are indicated on the left. (B) Cell lysates were immunoprecipitated with anti-myc antibody and analyzed by Western blotting (WB) with rabbit anti-flag (top) or anti-myc (bottom) antibodies (Ab). Mouse IgG was used as a control. Input materials (10%) were used as a reference standard. Molecular size markers are indicated on the left. (C and D) Endogenous PIASy and E12 coimmunoprecipitation. Cell lysates from mouse MCs or glomerular lysates from Thy1 rat were immunoprecipitated with anti-PIASy antibody and control IgG. The presence of E12 in the immunoprecipitates was determined by Western blotting with E12 antibody.

To determine whether the interaction of PIASy and E12 proteins observed in our yeast two-hybrid screen also occurs in mammalian cells, co-immunoprecipitation assays were performed. First, flag epitope-tagged PIASy and myc epitope-tagged E12 were cotransfected into COS7 cells. Cell lysates were then subjected to immunoprecipitation with anti-flag antibody followed by Western blotting with anti-myc antibody. We showed the presence of E12 in anti-flag immunoprecipitates, but not in control IgG precipitates based on Western blotting with anti-myc antibody (Figure 1A). Subsequent immunoprecipitation assays were performed with anti-myc antibody followed by Western blotting with anti-flag antibody (Figure 1B). Results showed that PIASy was detected in anti-myc immunoprecipitates. Furthermore, we demonstrated an association between endogenous PIASy and E12 in cultured mouse MCs by co-immunoprecipitation with anti-PIASy antibody, but not with control immunoglobulin G (Figure 1C). *In vivo*, we utilized an acute model of mesangial proliferative glomerulonephritis known as Thy1 glomerulonephritis (Thy1 GN) to examine whether observed interaction occurs between partner proteins expressed from endogenous genes in rat glomeruli. We also demonstrated an association between these proteins at day 6 by co-immunoprecipitation with anti-PIASy antibody, but not with control immunoglobulin G; however we did not detect the association at day 0 (Figure 1D). These results indicate that the interaction of PIASy and E12 could be dependent on mesangial activation.

### PIASy Blocked the Enhancement of α-SMA Promoter Activity Induced by E12

**Figure 2:**
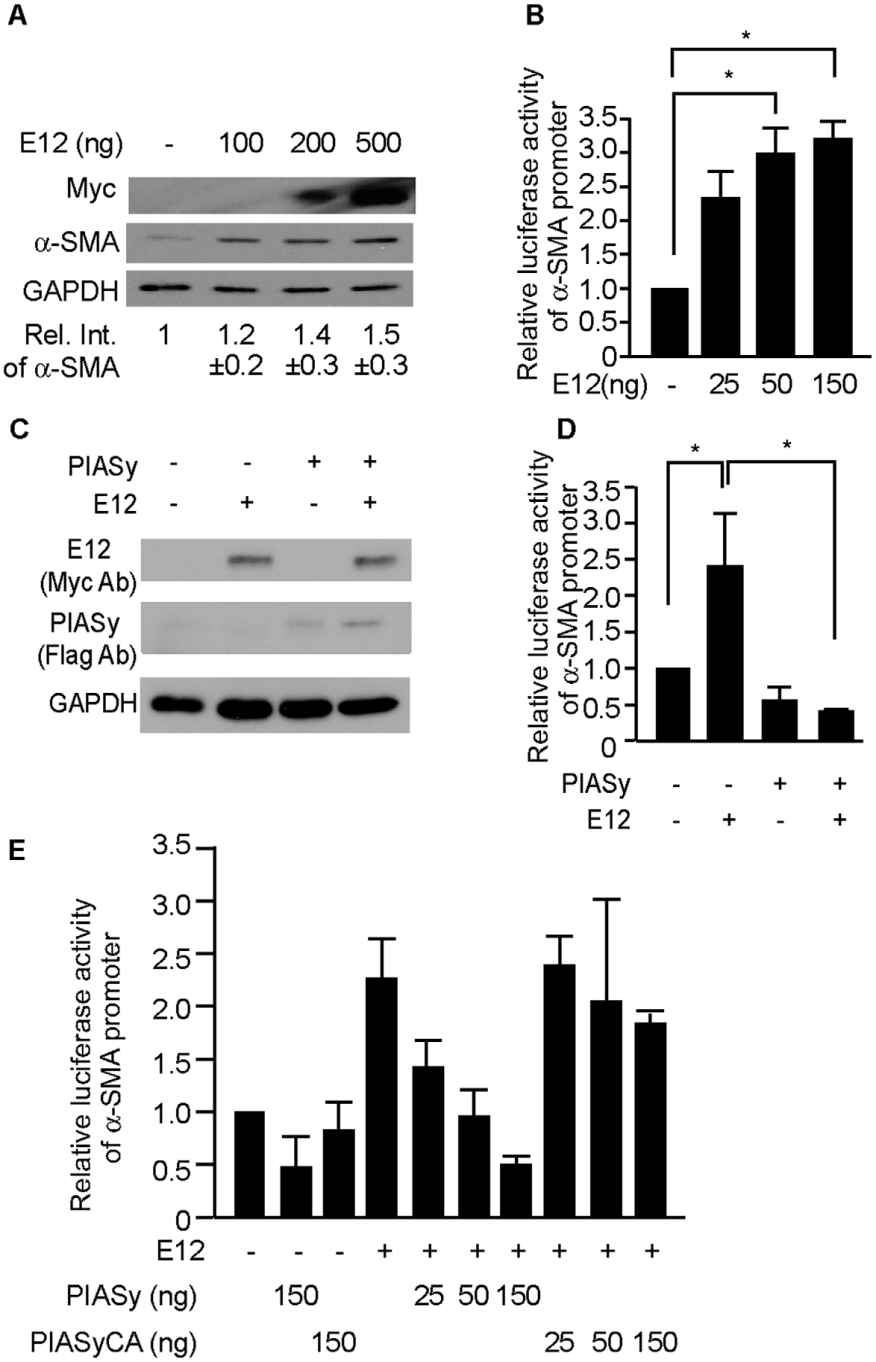
Effect of E12 and PIASy on α-SMA gene expression. (A) Effect of E12 on α-SMA gene expression. Mouse MCs (0.25×10^5^) were plated in 12-well plates, and six hours later, transfected with the E12 expression plasmid (100, 200, 500 ng plasmid DNA/well). After 48 hours, cell lysates were prepared, and α-SMA was visualized by Western blot analysis. Results are means ± SD (n = 5). (B) Mouse MCs (0.15×10^5^) were plated in 24-well plates, and six hours later, cotransfected with 25, 50, and 150 ng of the E12 expression plasmid, and 150 ng of the reporter construct. Luciferase activities in lysates prepared 36 hours post-transfection were measured. The activity of the reporter plasmid alone was arbitrary given a value of 1, and the activities of the other transfections were adjusted relative to this assay. Means of three independent experiments in triplicate are shown. (C) Mouse MCs were cotransfected with 150 ng of the PIASy expression plasmid, 25 ng of the E12 expression plasmid, and 150 ng of the reporter construct. Luciferase assay was performed as described in the legend for B. (D) Extracts from mouse MCs transfected as in C were prepared for Western blotting with anti-myc, anti-flag, and anti-GAPDH antibodies (Ab). (E) Mouse MCs were cotransfected with 25 ng of the E12 expression plasmid, and 150 ng of the reporter construct, together with or without increasing amounts of PIASy or PIASy^CA^ as indicated. Luciferase assay was performed as described in the legend for B. *P<0.05, v.s. control.

To examine whether E12 affects α-SMA gene expression, we tested the transcriptional activity of the α-SMA promoter reporter gene by co-transfecting with E12 expression plasmids to mouse MCs. Luciferase activity of the α-SMA promoter reporter gene was increased by co-transfection of E12 expression plasmids in a dose-dependent manner (Figure 2B), which was confirmed by Western blot analysis (Figure 2A). Then, to determine the effects of PIASy on α-SMA promoter activities, mouse MCs were co-transfected with PIASy and E12 expression plasmids along with the α-SMA promoter reporter gene. As shown in Figure 2D, E12 markedly increased the α-SMA promoter activity, whereas co-transfection of PIASy blocked the E12-induced increase. PIAS family members possess E3-ligase activity for SUMO (small ubiquitin-related modifier), and the RING domain of the PIAS protein is essential for this sumoylation. To examine the involvement of the RING domain on PIASy-mediated suppression of E12 activity, we used a PIASy RING mutant (PIASy^CA^), which was shown to have no sumoylation activity on Tcf-4 [14]. As shown in Fig. 2E, PIASy, but not PIASy^CA^ suppressed α-SMA activity induced by E12. These results suggest that sumoylation of E12 by PIASy through the RING domain is important for the PIASy-mediated suppression of α-SMA activation by E12.

### PIASy Acts as E3 Ligases for E12 Sumoylation

**Figure 3:**
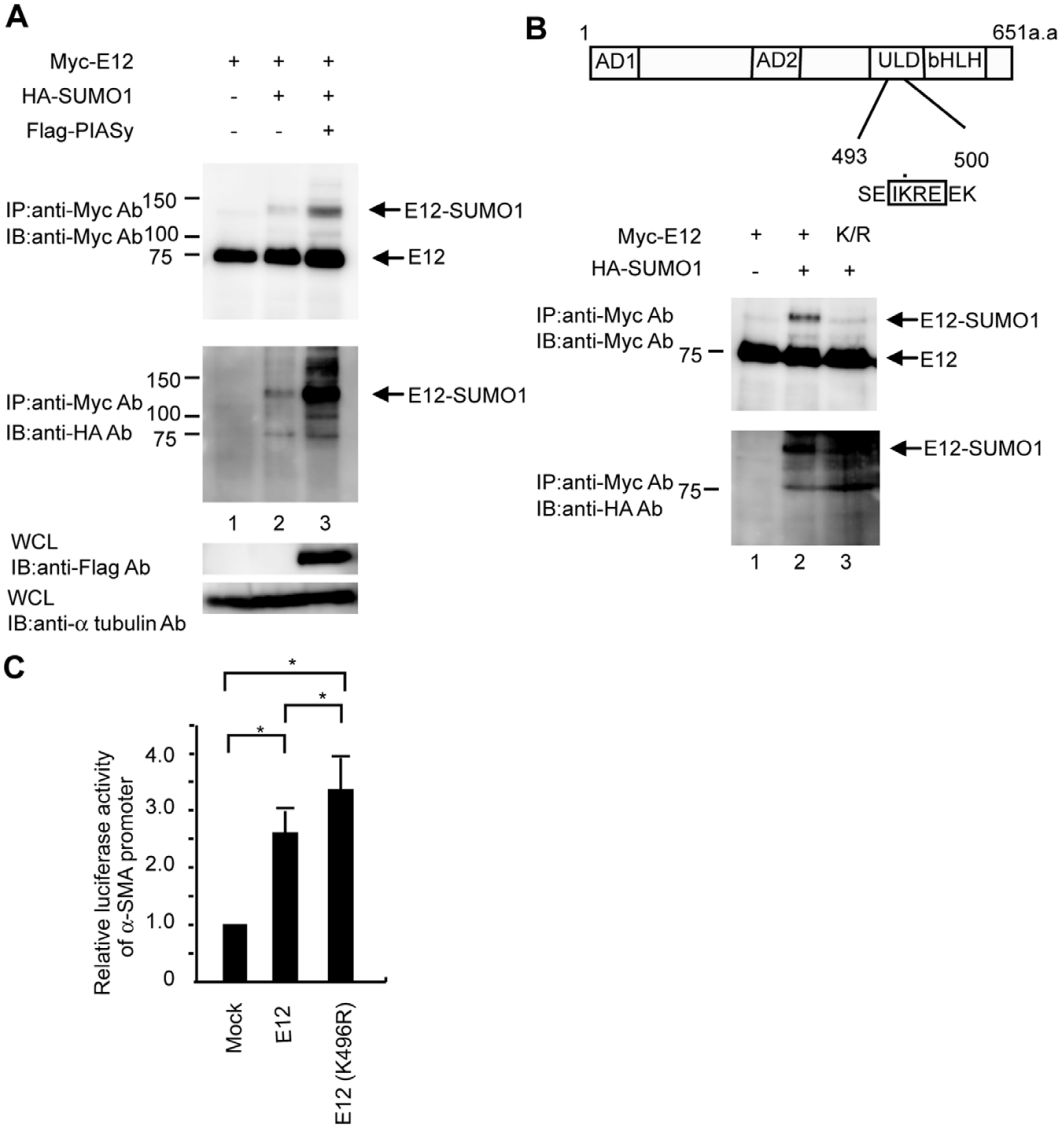
PIASy promotes sumoylation of E12 *in vivo*. (A) 293T cells were cotransfected with (+) or without (−) 2 µg of plasmid expressing myc-E12, 2 µg of plasmid expressing HA-SUMO-1, and 2 µg of plasmid expressing flag-PIASy. Cell lysates were immunoprecipitated (IP) with anti-myc antibody. The immunoprecipitates were subjected to SDS-PAGE and analyzed by Western blotting (WB) with anti-myc antibody. After ECL development, the filter shown in the first panel was stripped and reproved with anti-HA antibody (second panel). The levels of proteins expressed in whole cell lysates (WCL) were analyzed and shown as indicated. Each experiment was performed at least three times and representative data was shown. (B) SUMO-1 is conjugated at lysine 496 of E12 in vivo. Upper panel, schematic representation of mouse E12. The activation domain I (AD I), activation domain II (AD II) and ubiquitin ligase domain (ULD) are shown. The lysine residue in the putative SUMO-1 acceptor site is indicated by a black dot within the consensus sumoylation sequence. Lower panel, sumoylation of wild-type E12 but not mutant E12 (K/R) in vivo. 293T cells were transfected with plasmids expressing myc-E12 or myc-E12 (K/R) with (+) or without (−) HA-SUMO1. Cell lysates were immunoprecipitated (IP) with anti-myc antibody. The immunoprecipitates were subjected to SDS-PAGE and analyzed by Western blotting(WB) with anti-myc antibody. After ECL development, the filter shown in the first panel was stripped and reproved with anti-HA antibody (second panel). (C) Mouse MCs (0.15×10^5^) were plated in 24-well plates, and six hours later, cotransfected with 25 ng of the wild-type E12 or mutant E12 (K/R) expression plasmid, and 150 ng of the reporter construct. Luciferase activities in lysates prepared 36 hours post-transfection were measured. The activity of the reporter plasmid alone was arbitrary given a value of 1, and the activities of the other transfections were adjusted relative to this assay. Means of three independent experiments in triplicate are shown.

To determine whether E12 is modified by SUMO-1, *in vivo* sumoylation assays were performed by transiently expressing myc-tagged E12 and HA-tagged SUMO-1. Western blot analysis using anti-myc antibody revealed the presence of myc-tagged E12 in cells transfected with the plasmid expressing myc-E12. When HA-SUMO-1 was co-expressed, an additional slower migrating band was detected by anti-myc antibody, and anti-HA antibody identified the slower migrating form of E12 (Figure 3A, lane 2). These results suggest that SUMO-1 is conjugated to E12. Next, we examined the effect of PIASy expression on SUMO-1 modification of E12 in 293T cells. Sumoylation of E12 was enhanced by PIASy (Figure 3A, lane 3), indicating that PIASy is targeting E12 for the SUMO-1 modification.

E12 has a consensus sumoylation sequence, IKRE, which is conserved among other species (Figure 3B). We speculated that Lys-496 in mouse E12 is a likely target for sumoylation. To address this hypothesis, mutant E12 (K/R), in which lysine 496 was converted to Arg was prepared. Consistent to the previous observation, wild-type E12 was sumoylated *in vivo*; however, the transfection of E12 (K/R) abrogated the slower migrating band (Figure 3B), suggesting that Lys-496 is a major SUMO-1 conjugation site.

Sumoylation has been shown to affect the activity of many transcription factors. Therefore, we investigated whether sumoylation site mutations with lysine to arginine (K/R) affect α-SMA transcriptional activity. To test this, mouse MCs were transiently co-transfected with expression plasmids encoding wild-type E12 or mutant E12 (K/R), together with the α-SMA promoter reporter gene. As shown in Figure 3C, mutant E12 (K/R) showed significantly higher activity compared to that of the wild-type, suggesting that the sumoylation of E12 antagonizes its transcriptional activation potential.

### siRNA Specific for PIASy Increased the Transcriptional Activity of α-SMA in Cultured Mesangial Cells

**Figure 4:**
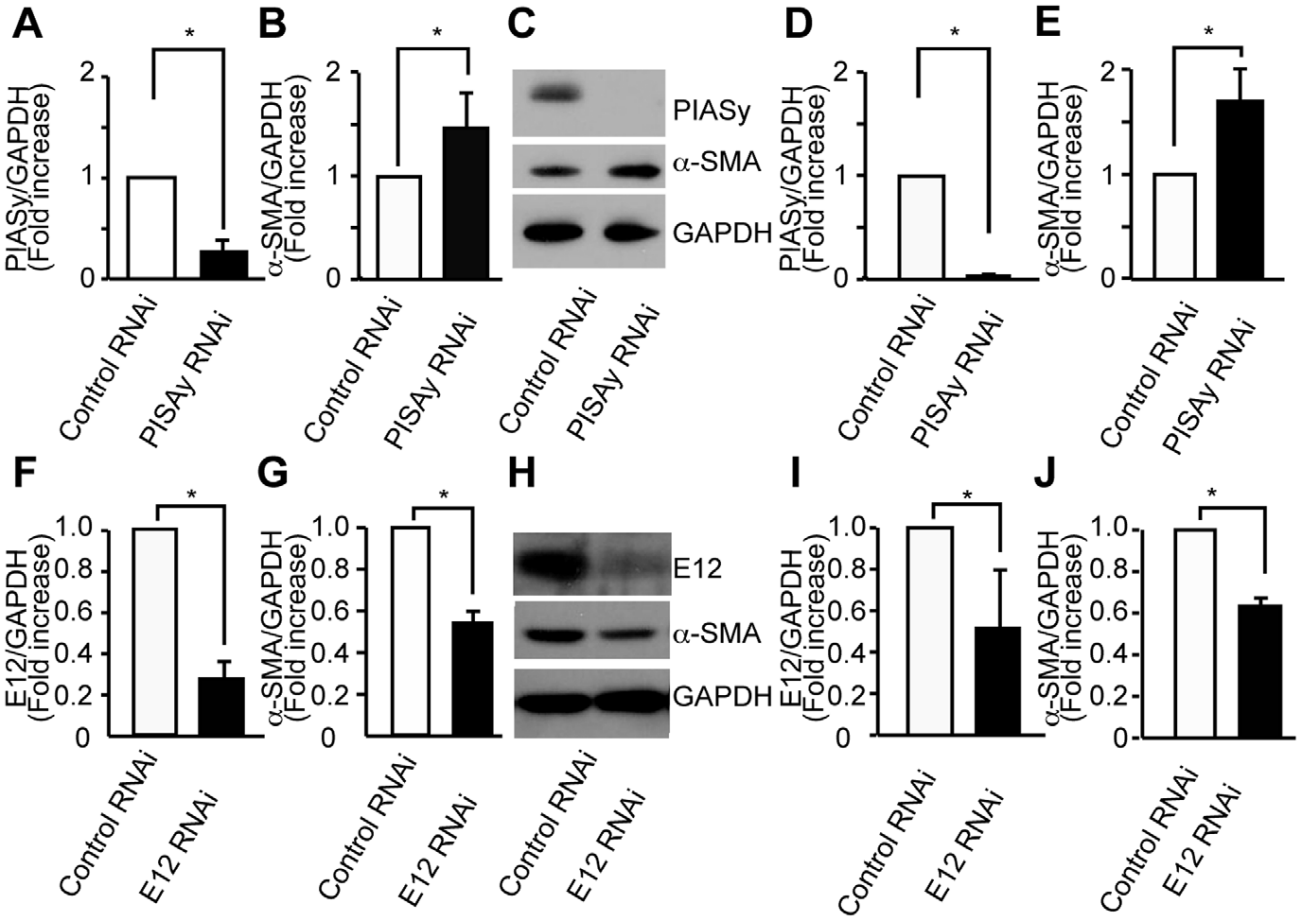
Effect of siRNA specific for PIASy and E12 in α-SMA regulation in MCs. (A) PIASy mRNA and protein were reduced by transfection of siRNA against PIASy in mouse MCs. Gene expression of PIASy was examined by quantitative RT-PCR using mRNA of MCs transfected with siRNA against PIASy or control siRNA (20 nM). (B) **α-SMA** mRNA expression was increased by transfection of siRNA against PIASy. (C) Western blots of MCs transfected with siRNA against PIASy or control siRNA. (D and E) Optical densitometry of PIASy and α-SMA in immunoblotting. (F) E12 mRNA and protein were reduced by transfection of siRNA against E12 in mouse MCs. Gene expression of E12 was examined by quantitative RT-PCR using mRNA of MCs transfected with siRNA against E12 or control siRNA (10 nM). (G) α-SMA mRNA expression was suppressed by transfection of siRNA against E12. (H**)** Western blot analysis of MCs transfected with siRNA against E12 or control siRNA. (I and J) Optical densitometry of E12 and α-SMA in immunoblotting. Means of four independent experiments are shown. The results were presented as the fold-increase or decrease compared with the values of cells transfected with control siRNA. GAPDH, glyceraldehyde-3-phosphate dehydrogenase. *P<0.05, v.s. control.

To address the role of endogenous PIASy and E12 in the regulation of α-SMA, we used stealth small interfering RNA (siRNA) to reduce the expression of PIASy and E12. MCs transfected with siRNA oligonucleotide against PIASy and E12 or control siRNA were harvested and analyzed by RT-PCR at 48 hours after transfection. siRNA for PIASy significantly increased endogenous α-SMA mRNA expression (Figure 4A and 4B), while siRNA for E12 reduced its expression in MCs (Figure 4F and 4G). In Western blot analyses, siRNA for PIASy significantly increased endogenous α-SMA expression (Figure 4C, D, E), while siRNA for E12 reduced its expression in MCs (Figure 4H, I, J).

### TGF-β Increased PIASy and E12 in Mesangial Cells

**Figure 5:**
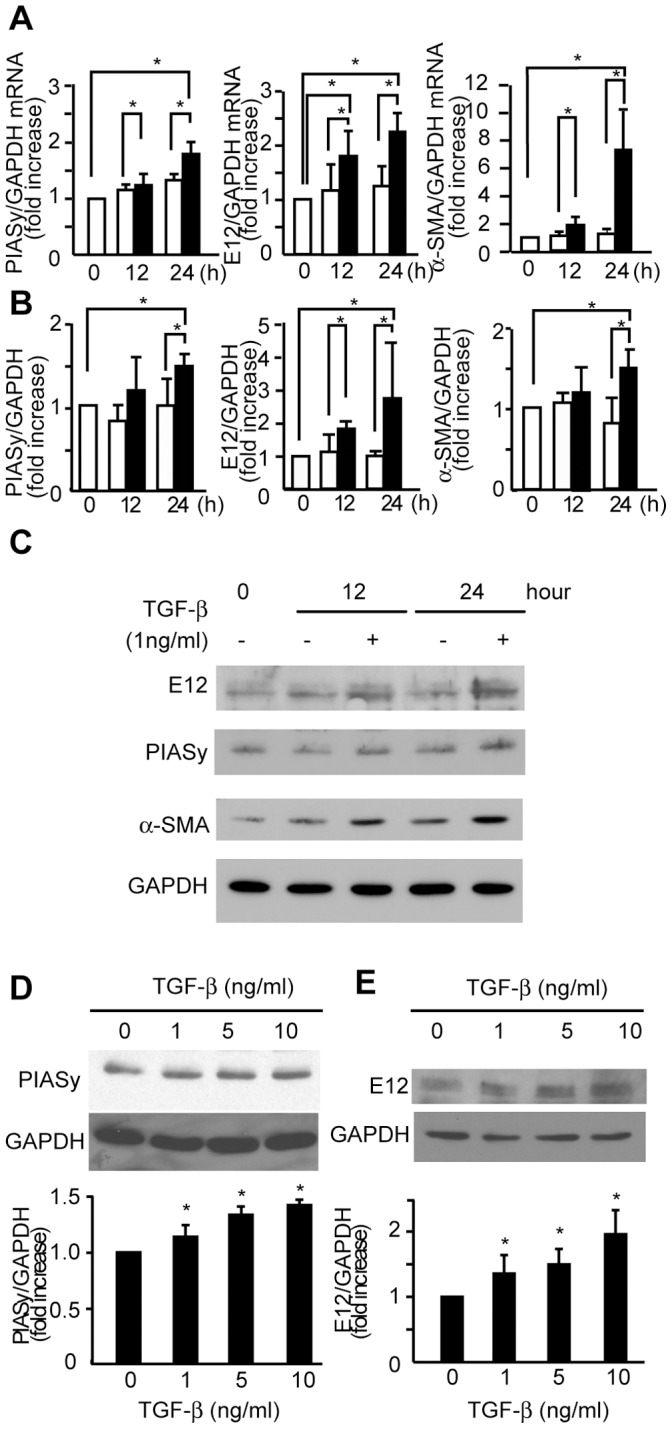
TGF-β increased PIASy, E12, and α-SMA mRNA and proteins in MCs. **(**A) Mouse MCs were cultured in the presence or absence of 1 ng/ml TGF-β for the indicated times. Gene expression of PIASy, E12 and α-SMA was examined by quantitative RT-PCR using mRNA of MCs treated or untreated with TGF-β. Means of four independent experiments are shown. The results are presented as the fold-increase or decrease compared with the values of 0 hours. *Open bars* denote no treatment, *closed bars* TGF-β treatment. (C) Mouse MCs were cultured in the presence or absence of 1 ng/ml TGF-β for the indicated times. Total cell lysates were examined by Western blot analysis using anti-PIASy, anti-E12, and anti-α-SMA antibodies. Representative data from four independent experiments is shown. (B**)** Optical densitometry of PIASy, E12 and α-SMA in Western blotting. The values of PIASy, E12 and α-SMA were normalized for that of GAPDH and compared with the values of 0 hours. Means of four independent experiments are shown. *Open bars* denote no treatment, *closed bars* TGF-β treatment. (D) TGF-β increased PIASy proteins in MCs in a dose-dependent manner. Mouse MCs were cultured with the indicated doses of TGF-β for 24 hours. (E) TGF-β increased E12 proteins in mesangial cells in a dose-dependent manner. Mouse MCs were cultured with the indicated doses of TGF-β for 24 hours. The values of PIASy and E12 were normalized for that of GAPDH and compared with the values of control vehicle. Means of four independent experiments are shown. *P<0.05, v.s. control.

The importance of TGF-β as a potent stimulus for phenotypic modulation of MCs has been demonstrated in previous reports [15], [16]. Therefore, we examined the effects of TGF-β on mRNA levels of PIASy and E12. Total RNA from MCs treated with 1 ng/ml TGF-β were analyzed by RT-PCR. We found that the levels of PIASy and E12 mRNA were significantly increased at 24 hours after TGF-β treatment in accordance with the increase of α-SMA (Figure 5A). TGF-β also increased PIASy and protein levels in MCs at 24 hours along with the increase of α-SMA (Figure 5B and C). Furthermore, the induction of PIASy and E12 proteins was increased dose dependently in response to TGF-β (Figure 5D and E).

### Effect of siRNA Specific for PIASy on TGF-β Mediated α-SMA Expression in Mesangial Cells

**Figure 6:**
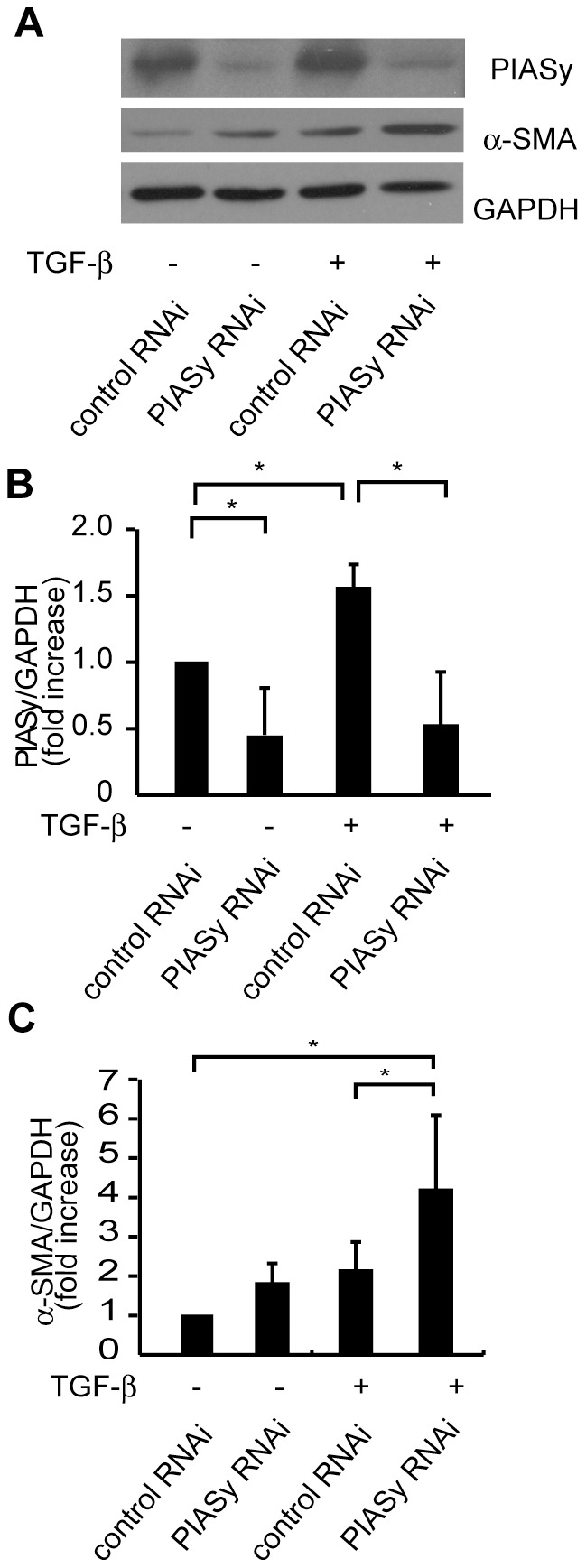
Effect of siRNA specific for PIASy on TGF-β mediated α-SMA expression in mesangial cells. (A) Mouse MCs were transfected with siRNA against PIASy or control siRNA. 12 hours after the transfection, MCs were serum-starved in starving medium (0.5% bovine serum albumin/DMEM) for 36 h. MCs were then stimulated with TGF-β (1 ng/ml) for 24 hours. Total cell lysates were examined by Western blot analysis using anti-PIASy and anti-α-SMA antibodies. Representative data from three independent experiments is shown. (B and C**)** Optical densitometry of PIASy and α-SMA in Western blotting. The values of PIASy, E12 and α-SMA were normalized for that of GAPDH and compared with the values of cells transfected with control siRNA. GAPDH, glyceraldehyde-3-phosphate dehydrogenase. *P<0.05, v.s. control.

To examine the role of PIASy in TGF-β mediated α-SMA expression, knockdown studies were done using siRNA specific for PIASy. In immunoblot analyses, PIASy was markedly reduced in cells transfected with siRNA compared with that transfected with control RNAi in both TGF-β treated or non-treated cells (Figure 6A and B). The expression of α-SMA protein after 24 hours of stimulation with TGF-β was markedly increased by siRNA for PIASy, indicating that TGF-β up-regulated PIASy expression in MCs to down-regulate α-SMA gene transcription (Figure 6A and C).

### Expression of α-SMA TGF-β and E12 in Experimental Mesangial Proliferative Glomerulonephritis

**Figure 7:**
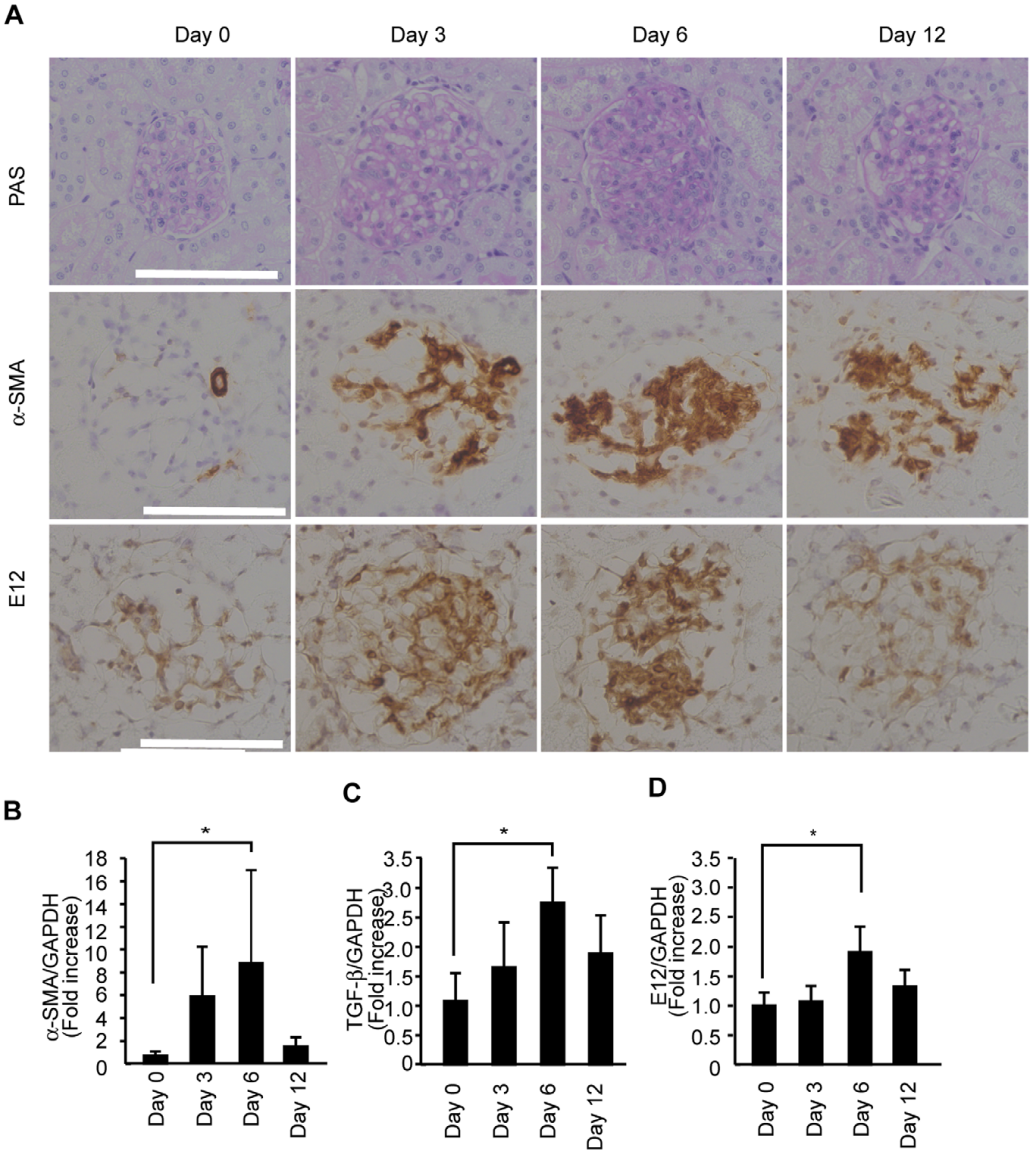
Glomerular expression of E12, α-SMA, and TGF-β in Thy1 GN. (A**)** Representative light microscopic pictures of glomeruli (periodic acid-Schiff staining) and expression of α-SMA and E12 at day 0, 3, 6 and 12 in Thy1 GN. Significant proliferation of MCs was observed at day 6. (B, C and D) Gene expression of E12, α-SMA, and TGF-β was examined by quantitative RT-PCR using glomerular mRNA. The results were presented as the fold increase compared with the values obtained before the induction of Thy1 GN (day 0). GAPDH, glyceraldehyde-3-phosphate dehydrogenase. *P<0.05, v.s. control. White scale bar = 100 µm. Original magnifications, ×400.

We utilized an acute model of mesangial proliferative glomerulonephritis known as Thy1 glomerulonephritis (Thy1 GN) to examine whether PIASy and E12 are up-regulated along with TGF-β and α-SMA *in vivo*. In Thy1 GN, the proliferation of MCs began at day 2, peaked at day 6, and subsided at 12 days after the injection. Figure 7A shows a representative light microscopic picture at days 0, 3, 6, and 12. Immunohistochemical analysis revealed that α-SMA was hardly seen in glomeruli before induction of Thy1 GN, but was highly expressed at day 6 (Figure 7A). In parallel to the α-SMA expression, E12 was slightly seen before the induction, but was highly expressed in the glomeruli at day 6 (Figure 7A). To analyze the development of Thy1 GN, we examined glomerular gene expression of TGF-β, α-SMA and E12 by quantitative RT-PCR, and found that TGF-β, α-SMA, and E12 mRNA were increased at day 6 in a parallel fashion (Figure 7B, C and D).

### PIASy and SUMO-1 Expression in Experimental Mesangial Proliferative Glomerulonephritis

**Figure 8:**
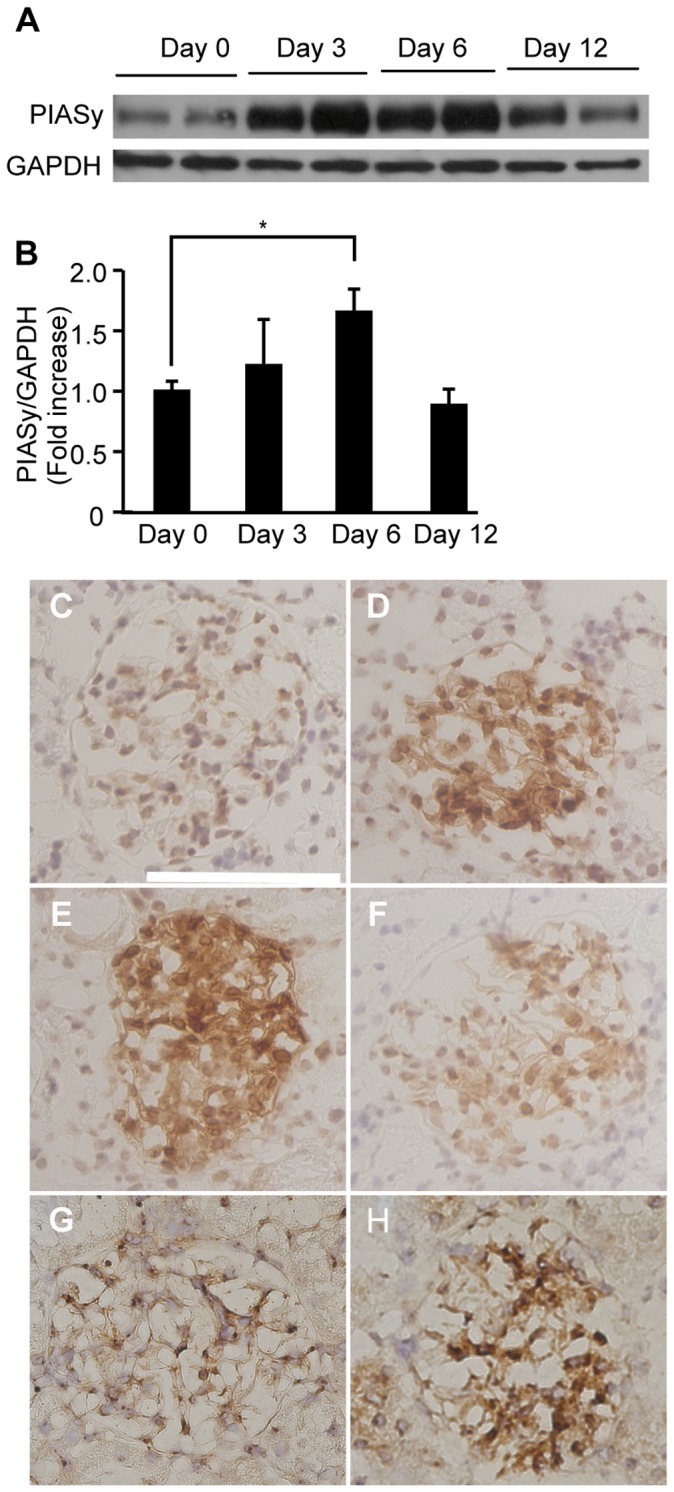
Up-regulation of PIASy and SUMO-1 in Thy1 glomerulonephritis. (A) Expression of PIASy protein in glomeruli from Thy1 GN. Glomerular lysates were subjected to Western blot analyses with an anti-PIASy antibody. Representative picture is shown. PIASy was significantly upregulated at day3 and 6 in the course of Thy1 GN (n = 4; P<0.05) (B) Optical densitometry of PIASy in Western blotting. The data are expressed as mean ± SD (n = 4 in each group). (C, D, E and F) Immunohistological analysis of PIASy in Thy1 glomerulonephritis at day 0 (C), day 3 (D), day 6 (E) and day 12 (F). Note that nuclear staining of PIASy was seen in the glomeruli at day 3 and 6. (G and H) Immunohistological analysis of SUMO-1 in Thy1 glomerulonephritis at day 0 (G), and day 6 (H). Sections were counterstained with hematoxylin solution. White scale bar = 100 µm. Original magnifications, ×400.

Glomerular expression of PIASy was then examined by Western blotting and immunohistochemical analysis. Figure 8A shows that PIASy protein was significantly increased at day 6. Immunohistochemical analysis confirmed that PIASy was expressed at a low level in glomeruli before induction, and its expression was dramatically induced at day 6 (Figure 8E). We noticed that PIASy and E12 were upregulated and distributed similarly in glomeruli in the proliferative phase. Moreover, immunohistological analysis revealed that SUMO-1 was also distributed similarly in glomeruli at day 6, suggesting that protein sumoylation was involved in the progression of glomerulonephritis (Figure 8G and H).

### PIASy Inhibited Cell Proliferation in MCs

**Figure 9:**
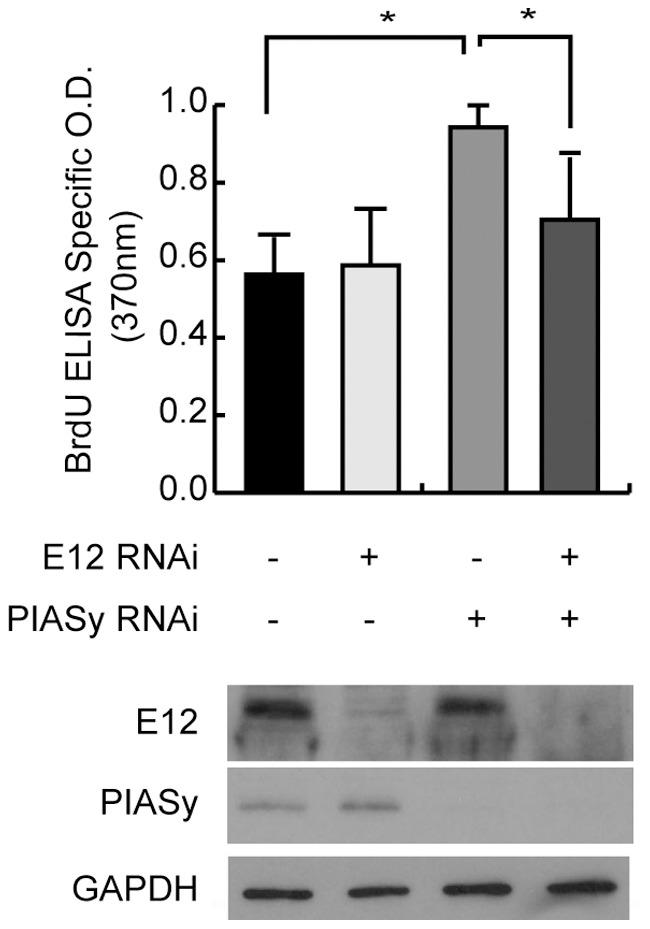
Effect of siRNA specific for PIASy and E12 in cell proliferation in MCs. siRNA specific for E12 did not affect the BrdU incorporation, but siRNA specific for PIASy obviously enhanced the cell proliferation 48 hours later after the transfection. The enhancing effect was significantly attenuated in combination with the knockdown of E12 by the RNA interference. Values were expressed as means ± SD (n = 3).

Finally, we determined if E12 and PIASy were involved in cell proliferation in MCs. Knockdown of E12 alone by siRNA did not affect the BrdU incorporation, whereas knockdown of PIASy significantly enhanced the cell proliferation at 48 hours after transfection. Moreover, the enhancing effect of siPIASy was significantly attenuated when we knocked down E12 at the same time (Figure 9). These results indicate that PIASy plays an important role in cell proliferation of MCs through the interaction with E12.

## Discussion

In this study, we have identified an association between PIASy and E12 and demonstrated the role of PIASy as a negative regulater of α-SMA expression in MCs upon the interaction with E12. Furthermore, at least part of its activity is dependent on E12 sumoylation by PIASy. TGF-β treatment increased PIASy and E12 in accordance with α-SMA induction. Moreover, reduced expression of PIASy protein by siRNA specific for PIASy leads to an increase in the magnitude of TGF-β mediated α-SMA expression. We also demonstrated that the expression of PIASy and E12 was markedly increased in the proliferative phase in Thy1 GN concomitant with α-SMA expression, suggesting that PIASy and E12 are involved in the regulation of α-SMA.

In the present study, the α-SMA gene is regulated by E12 in MCs, suggesting that E12 functions as a transcription factor. Furthermore, PIASy negetively regulates α-SMA expression upon the interaction with E12. Interestingly, Kawai-Kowase et al reported that PIAS1 induces transcriptional activation of SMC differentiation marker genes through cooperative interactions with both SRF and class1 bHLH proteins in cultured smooth muscle cells [17]. This difference might be derived from that PIAS proteins exert differential effect on E12. Alternatively, tissue-specific factors may modulate the PIAS function.

Moreover, we found that disruption of the E3-ligase activity of PIASy abolished its ability to down-regulate the α-SMA promoter, suggesting that at least part of its activity is dependent on protein sumoylation. PIAS family proteins have been originally identified as a cofactor that inhibits the transcriptional activation potential of STAT and in mammals five PIAS proteins (PIAS1, 3, Xα, Xβ, and y) have been reported [18]. Recently, PIAS family proteins have been proposed to function as a SUMO-E3 ligase [19]. PIASy was shown to catalyze sumoylation of p53, LEF-1, Smad3, C/EBPδ, E1AF, Ets-1 and Tcf-4 [14], [20], [21], [22], [23], [24], [25]. Intriguingly, we found that PIASy acts as E3 ligases for E12 sumoylation by *in vivo* sumoylation assays. Moreover, we found that PIASy preferably mediates SUMO-1 modification of E12 over SUMO-3 modification (Figure S2). SUMO-1 modification and SUMO-2/3 modification can result in distinct consequences in alteration of target protein’s function. Further study is required to address the role of SUMO modification in the function of E12 and its modification by PIASy.

Sumoylation has been shown to affect the activity of many transcription factors. We found that mutant E12 (K/R), E12 sumoylation defective mutants, showed significantly higher α-SMA promoter activity compared to that of the wild-type, suggesting that the sumoylation of E12 antagonizes its transcriptional activation potential. Next, we examined whether overexpressing PIASy has a different effect on E12 (either wild-type or K/R)–mediated transactivation. As shown in Figure S3, the percentage of decrease in mutant E12 (K/R)-mediated transactivation by overexpressing PIASy was significantly lower than that of the wild-type, suggesting that mutant E12 is more resistant to the effect of PIASy overexpression.

Due to the significant sequence homology within the PIAS family and their redundant interactions, it is likely that other post-transcriptional modifications and/or their local concentrations largely govern their specificity *in vivo*. As shown in Figure S1B, *in vivo* sumoylation assay revealed that PIAS1 also acts as E3 ligases for E12 sumoylation in 293T cells, but the minor slowly migrating bands were weaker than in the case of PIASy. On the other hand, we examined the expression level of PIAS family members in mouse MCs. As shown in Figure S1A, RT-PCR revealed that PIASy is most predominantly among PIAS family members expressed in mouse MCs. These results suggest that PIASy predominantly acts as E3 ligases for E12 in MCs among PIAS family members.

In this study, we showed that TGF-β transcriptionally increased the expression of PIASy and E12 along with α-SMA expression in MCs. Moreover, reduced expression of PIASy protein by siRNA specific for PIASy resulted in increased TGF-β-mediated α-SMA expression. These results suggest that PIASy negatively regulates TGF-β signaling by associating with E12 to prevent uncontrolled cell proliferation and dedifferentiation, although this is not the only mechanism involved in the control of this signal transduction. Imoto et al demonstrated that PIASy associates with Smad3 and inhibits TGF-β/Smad transcriptional responses such as PAI-1 using the negative feedback loop [20]. Thus it is also possible that the effect of PIASy on TGF-β signaling is mediated through multiple cascades.

In Thy1 GN, PIASy and E12 were upregulated and distributed similarly in the nucleus of the glomerulus in the proliferative phase, along with increased α-SMA and TGF-β expression, suggesting that these genes were involved in the regulation of α-SMA under pathophysiological conditions *in vivo*, and that tight control of HLH levels and activity by PIASy is necessary to prevent uncontrolled cell proliferation and dedifferentiation. Interestingly, an association between PIASy and E12 proteins was observed at day 6 by IP-western blotting, but not at day 0, suggesting that the interaction of PIASy and E12 could be dependent on mesangial activation. Taken together, we speculated that TGF-β up-regulates PIASy expression in MCs at day 6 to regulate α-SMA gene transcription by the interaction with E12. In this model, however, we could not conclude whether PIASy down-regulates α-SMA expression as demonstrated in *in vitro* knockdown assays. Future studies using PIASy-deficient mice are needed.

In this study, we focused on E-box dependent α-SMA transactivation. On the other hand, several key transcription factors have been identified and shown to be important in regulation of TGF-β-induced SMC-specific gene expression, including SRF [26] and Smad family [27]. This may be one reason why PIASy was increased in accordance with α-SMA expression at day 6 in Thy1 GN. However, in view of the homeostatic importance of TGF-β, the targeting modalities would not need to interfere with physiological activity. Partial attenuation of overactivity appears to be sufficient to limit the consequences on tissues. Prevention of diabetic retinal microangiopathy did not require complete normalization of gene expression changes in the TGF-β pathway [28]. Interestingly, siRNA specific for PIASy significantly increased endogenous type 1 collagen (1.24±0.2 fold) and PAI-1 (1.31±0.32 fold) mRNA expression, suggesting that PIASy may be involved not only in α-SMA expression but also in phenotypic changes of MCs. Moreover, we found that PIASy plays an important role in mesangial cell proliferation through the interaction with E12 (Figure 9). These results suggest that PIASy may be a therapeutic target in glomerulonephritis.

In summary, we show that PIASy is a novel E12-binding partner, and enhances its sumoylation as a specific SUMO-E3 ligase. Our study indicates that TGF-β up-regulates PIASy expression in MCs to down-regulate α-SMA gene transcription by the interaction with E12. Further analysis of the modulation of E12-induced α-SMA expression by PIASy is likely to provide us new insights into molecular pathophysiology of progressive renal disease and helps establish new therapeutic approaches to glomerulosclerosis.

## Materials and Methods

### Reagents and Antibodies

Human recombinant TGF-β1 was purchased from R&D Systems (Minneapolis, MN). Rabbit anti-rat PIAS4, anti-E12 (Santa Cruz Biotechnology, Santa Cruz, CA), Rabbit anti-mouse PIAS4 (Proteintech Group, Chicago, IL), anti-α-SMA (1A4), Rabbit anti-flag, mouse anti-flag M2 (Sigma-Aldrich,St Louis, MO), anti-GAPDH (6C5; Millipore, Billerica, MA), rat anti-HA (3F10; Roche), and mouse anti-α-tubulin (Ab-1; Oncogene) were purchased commercially.

### Yeast two-hybrid Screening

Yeast two-hybrid screening was performed with a Matchmaker GAL4 two-hybrid system (Clontech, Mountain View, CA) using the reporter Saccharomyces cerevisiae strain AH109 as described by the manufacturer. To generate a bait construct with the bHLH domain of E12 (505–651 aa), the cDNA was amplified by PCR from the full-length cDNA mouse E12 (RIKEN), and inserted into the NcoI-PstI site of the pGBKT7 vector. We prepared cDNA library from mouse mesangial cells and inserted it into the pGADT7-Rec vector. Primary screening was based on activation of the histidine selection marker by an interaction between bait and library proteins and was performed using histidine-negative plates. Secondary screening was based on further activation of a β-galactosidase reporter gene and was determined using blue/white colony screening. Library-derived plasmids from the candidate clones were rescued into the E. coli DH-5α and studied further.

### Cell Cultures

Human embryonic kidney carcinoma cell line, 293T, and COS7 cells were maintained in Dulbecco’s modified Eagle’s medium (DMEM) supplemented with 10% fetal bovine serum and glutamine as previously described [29], [30]. Mouse kidney mesangial cells (MCs) were characterized and maintained in DMEM supplemented with 20% fetal bovine serum and glutamine as previously described [31]. The cultured cells fulfilled the criteria generally accepted as glomerular MCs [32].

### Expression Plasmid Construction

pcDNA3-HA-SUMO-1 were kindly provided by Dr. K. Shimotohno [33]. pCAG-HA-SUMO-1, HA-SUMO-3 [34], pEGFP-PIAS1, pEGFP-PIASXα, pEGFP-PIASXβ, pEGFP-PIAS3 [35] and pEGFP-PIASy [36] were kindly provided by T. Ohshima. The fragment of mouse α-SMA (−1074 to +43) were subcloned into a pGL3-basic vector (Promega, Madison, WI) [37]. Full-length of mouse PIASy was amplified by PCR from the cDNA in mouse testis and cloned into the EcoR I/Xba I sites of pcDEF3-flag vector. Full-length of mouse E12 was amplified by PCR from the full-length cDNA mouse E12 (RIKEN) and cloned into the Sal I/Bgl II sites of PCMV-myc vector (Clontech). The cDNAs for mutant E12 with substitution of Lys-456 to Arg, E12 (K/R), and PIASy RING mutant with substitution of Cys-335 and Cys-340 to Ala, PIASy^CA^, were created using site-directed mutagenesis and subcloned into expression vectors to obtain pCMV-myc-E12 (K/R) and pcDEF3-flag-PIASy^CA^. All constructs were confirmed by DNA sequencing.

### Immunoprecipitation Experiments (IP-Western Blotting)

For the co-immunoprecipitation experiments, COS-7 cells were transfected with plasmid DNA using Fugene6 (Roche), according to the manufacturer’s instructions. Cells were harvested 48 hours after transfection, and lysed on ice in 20 mmol/L Tris-HCl (pH7.4), 150 mmol/L NaCl, 0.5% NP-40, supplemented with a protease inhibitor cocktail (Roche) for 20 minutes. The lysates were then precleared with protein agarose G (Roche) and incubated overnight at 4°C with anti-myc antibody (9E10, Upstate) followed by 3-hour incubation with protein agarose G. For immunoprecipitation with anti-flag antibody, the lysates were incubated overnight at 4°C with anti-flag M2 affinity gel (Sigma). Samples were washed four times with phosphate-buffered saline buffer, and immunoprecipitates were eluted and analyzed by Western blot.

For the endogenous proteins, mouse MCs lysates or rat glomerular lysates were lysed on ice in 20 mmol/L Tris-HCl (pH7.4), 150 mmol/L NaCl, 0.5% NP-40, supplemented with a protease inhibitor cocktail (Roche) for 20 minutes. The lysates were then precleared with protein agarose A (Roche) and incubated overnight at 4°C with anti-PIAS4 antibody (Cell Signaling Technology, Beverly, MA) followed by 3-hour incubation with protein agarose A. Samples were washed four times with phosphate-buffered saline buffer, and immunoprecipitates were eluted and analyzed by Western blot.

### Western Blot Analysis

MCs or glomerular lysates were lysed in RIPA buffer as described in previous reports [38]. Twenty µg of each sample was fractionated on SDS-PAGE gels and electroblotted onto Protran nitrocellulose membrane (Whatmann, UK), subjected to Western blot using each antibody. Immunoreactive bands were visualized and quantificated by imaging densitometer, Image J (http://rsb.info.nih.gov/ij/index.html).

### Reporter Assay

Transfections were perfomed using Fugene6. Luciferase activities were normalized to Renilla luciferase activities derived from cotransfected pRL-SV40-Luc (Promega). All reporter assays were performed in triplicate, and standard deviations (S.D.) are denoted by the bars in figures.

### *In vivo* Sumoylation Assays

HEK293T cells were transfected using Lipofectamine2000 (Invitrogen) according to the manufacturer’s instructions. *In vivo* sumoylation assays was performed as described previously [33].

### Preparation of Total RNA and Quantitative Reverse Transcription-PCR

Total RNA was isolated by TRIzol reagent (Invitrogen, Carlsbad, CA). The quantitative RT-PCR was performed as in previous reports [38]. The oligonucleotide primers are listed in Table 1.

**Table 1:** Primer sequences used in real time quantitative RT-PCR.

Gene	Forward	Reverse	GenBank Entry
Mouse and rat E12	ATACAGCGAAGGTGCCCACTT	AAAGGTGGCATAGGCATTCCG	AK017617
Mouse PIASy	CCACCAACCGCATTACTGTCA	TCACCCCAATCGTCTTCAACC	NM_021501
Mouse α-SMA	GCGTGAGATTGTCCGTGACAT	GCGTTCGTTTCCAATGGTGAT	NM_007392
Rat α-SMA	GGCATCCACGAAACCACCTAT	CCTTCTGCATCCTGTCAGCAA	NM_031004
Rat TGF-β	GCTGAACCAAGGAGACGGAAT	CGGTTCATGTCATGGATGGTG	NM_021578
Mouse and rat GAPDH	GCCTCACCCCATTTGATGTTA	GGCAAATTCAACGGCACAG	BC083149
Mouse Ubc9	GATGACTATCCGTCCTCACCACC	GGTGATAGCTGGCCTCCAGTCC	NM_011665.4
Rat Ubc9	AACCCTGATGGCACGATGA	CCCCTTCTTTCCAGGGATAGC	NM_013050.1

### RNA Interference

Stealth small interference RNA (siRNA) against E2A (5′-AAUACUGGGAGCUGCUCUUGAUGCC-3′), PIASy (5′-AAAGCUCUGGGCUACAGUCGAACUG-3′) and Stealth RNA Interference (siRNA) negative control duplex were provided by Invitrogen. Transient transfection of the siRNA oligonucleotides was carried out using Lipofectamine RNAi MAX (Invitrogen) according to the manufacturer’s instructions.

**Table 2:** Primer sequences used in RT-PCR.

Gene	Forward	Reverse	GenBank Entry	Product size (bp)
Mouse PIAS1	TCCTGCTGTAGATACAAGCTAC	TGCCAAAGATGGACGCTGTGTC	NM_019663	394
Mouse PIASX	GACTTTGCTTGGCAGAGACC	AAAGGGCACATCAAGGACAC	NM_008602	409
Mouse PIAS3	GTGGACATGCATCCTCCTCT	GCGTTCGTTTCCAATGGTGAT	NM_146135	405
Mouse PIASy	AGACCCTTAAGCCGGAGGTA	GTGGCCGAGGACAGATACAT	NM_021501	391

### Animals and Induction of Thy1 Glomerulonephritis

Male Wistar Kyoto rats (Shimizu Laboratory Animal Center, Hamamatsu, Japan) weighing 180–200 g were used in this study. Rats were housed under specific pathogen-free conditions. All animal experiments were performed in accordance with institutional guidelines, and the Review Board of Kyoto University granted ethical permission for this study (Permit Number: Med Kyo 09270). Thy1 GN was induced by a single intravenous injection of anti-rat Thy-1 monoclonal antibody (1 mg/kg) (Cedarlane Laboratories, Ontario, Canada) as described elsewhere [3]. These rats were sacrificed to obtain renal specimens, total glomerular RNA, and protein at days 3, 6 and 12 (n = 4 per group). Four rats were injected with vehicle only and sacrificed as controls. Rat glomeruli were isolated from renal cortex of rats using the differential sieving method [39], [40]. The purity of the glomeruli was >90%.

### Immunohistochemistry

Kidney halves were fixed in methyl Carnoy’s solution and embedded in paraffin. Sections (2 µm) were stained with periodic acid-Schiff for routine histology. For the immunohistochemistry, cryopreserved kidney tissues were cut in 4-µm-thick sections, fixed in acetone for 20 min, and treated with 0.3% H_2_O_2_ in methanol for 15 minutes. Sections were blocked with the appropriate preimmune serum for 60 min at room temperature, followed with primary antibodies anti-PIASy antibody (clone PIA4, Sigma-Aldrich), anti-E12 antibody (Santa Cruz), anti-α-SMA antibody (clone 1A4, Sigma-Aldrich), and anti-SUMO1 antibody (Zymed Laboratories, CA, USA).

### Reverse Transcription-Polymerase Chain Reaction

1 µg of total RNA was used to prepare complementary DNA (cDNA) with Superscript III reverse transcriptase (Invitrogen). 5 µl of cDNA was used as a template in the PCR reaction. PCR amplification was performed using Taq polymerase with primers. The oligonucleotide primers are listed in Table 2.

PCR was performed under the following conditions: 94°C for 2 minutes followed by 25 cycles at 94°C for 30 seconds, 50°C for 30 seconds, and 72°C for 1 minute, ending with a final extension at 72°C for10 minutes. The PCR products were run on 1% agarose gels and visualized by ethidium bromide staining.

### Cell Proliferation Assay with Transient Transfections of siRNA

BrdU ELISA was performed according to the manufacturer’s instructions. Briefly, 2500 mouse MCs/well were plated out in 96-well flat-bottomed microtiter plates in B medium/10% FCS. Six hours later, siRNA for PIASy, E12 and the control (Invitrogen) were transfected with Lipofectamine RNAi/MAX reagent (Invitrogen) according to the manufacturer’s instructions. The proliferation of MCs was determined at 48 hours after the siRNA transfections using a colorimetric immunoassay, based on the measurement of BrdU incorporation during DNA synthesis (Amersham Biosciences). BrdU was added to the medium for the final 2 h of treatment. Cells were incubated for 30 min with diluted, peroxidase-conjugated anti-BrdU antibody. Absorbance was assessed at 370 nm with 492 nm as the reference wavelength utilizing a microplate ELISA reader. Appropriate control wells were used in each experiment.

### Statistical Analysis

The data are expressed as the means ± SD. Comparison among more than two groups was performed by one-way analysis of variance followed by the post hoc analysis (Bonferroni/Dunn test) to evaluate statistical significance. All analyses were performed using StatView (SAS Institute, Cary, NC). Statistical significance was defined as P<0.05.

## Supporting Information

Figure S1 **Sumoylation of E12 is predominantly enhanced by PIASy among PIAS family members.** (**A**) 1 µg of total RNA from cultured mouse MCs or mouse testis was used to prepare complementary DNA (cDNA). PCR was done with oligonucleotide pairs for PIAS family members using 5 µl of cDNA. bp, base pairs. (B) 293T cells were cotransfected with 2 µg of plasmid expressing myc-E12 together with (+) or without (−), 2 µg of plasmid expressing HA-SUMO-1, and 2 µg of plasmid expressing GFP-PIAS1 (1), -PIAS3 (3), -PIASXα (Xα), -PIASXβ (Xβ) and -PIASy (y). Upper panel, Cell lysates were subjected to immunoblotting with anti-myc antibody. Middle and Lower panel, Cell lysates were immunoprecipitated (IP) with anti-myc antibody. The immunoprecipitates were subjected to SDS-PAGE and analyzed by Western blotting (WB) with anti-myc antibody. After ECL development, the filter shown in the middle panel was stripped and reproved with anti-HA antibody (lower panel).
(TIF)

Figure S2 **PIASy promotes SUMO-1 and SUMO-3 modification of E12 in vivo.** 293T cells were cotransfected with (+) or without (−) 2 µg of plasmid expressing myc-E12, 2 µg of plasmid expressing HA-SUMO-1 or HA-SUMO-3, and 2 µg of plasmid expressing flag-PIASy. Upper panel, Cell lysates were subjected to immunoblotting with anti-myc antibody. Middle and Lower panel, Cell lysates were immunoprecipitated (IP) with anti-myc antibody. The immunoprecipitates were subjected to SDS-PAGE and analyzed by Western blotting (WB) with anti-myc antibody. After ECL development, the filter shown in the middle panel was stripped and reproved with anti-HA antibody (lower panel).
(TIF)

Figure S3 **Effect of PIASy on mutant E12 (K/R)-induced α-SMA gene expression.** Mouse MCs (0.15×10^5^) were plated in 24-well plates, and six hours later, cotransfected with 50 ng of the PIASy expression plasmid, 25 ng of the wild- type E12 (WT) or mutant E12 (K/R) expression plasmid, and 150 ng of the reporter construct. Luciferase activities in lysates prepared 36 hours post-transfection were measured. Luciferase activities were normalized to Renilla luciferase activities derived from cotransfected pRL-SV40-Luc. The relative activities with wild-type E12 or mutant E12 (K/R) alone were designated as 100% (lanes 1 and 2). The percentage of decrease by overexpressing PIASy was compared between wild-type E12 and mutant E12. Results are the mean ± SD of data by taking the average of triplicates.
(TIF)
