# Supplementary figures and images for: Protein Inhibitor of Activated STAT, PIASy Regulates α-Smooth Muscle Actin Expression by Interacting with E12 in Mesangial Cells
Source: PLoS One. 2012 Jul 19;7(7):e41186. doi: 10.1371/journal.pone.0041186 (PMC3400623; doi:10.1371/journal.pone.0041186)

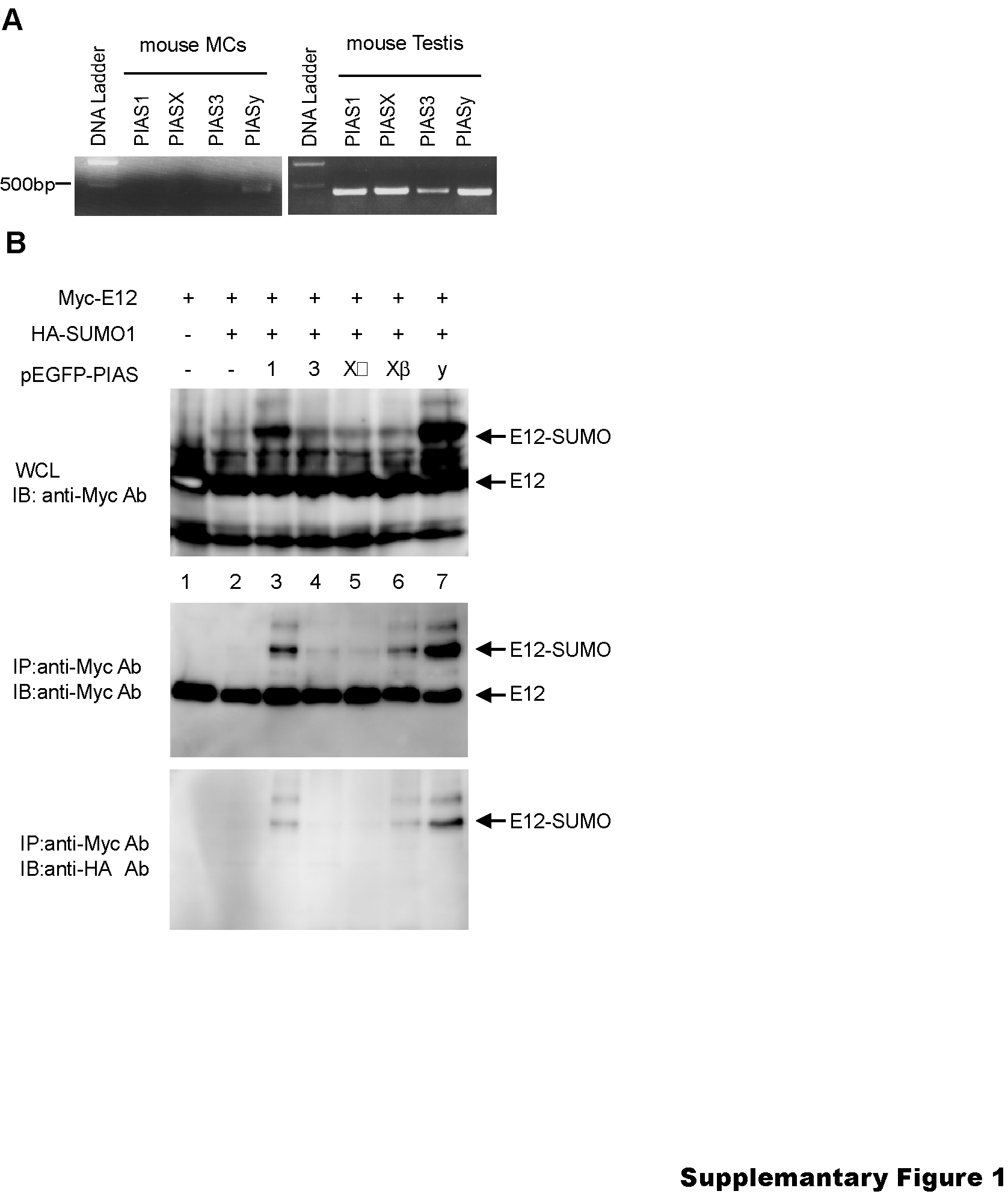

Supplement: Figure S1 — Sumoylation of E12 is predominantly enhanced by PIASy among PIAS family members. (A) 1 µg of total RNA from cultured mouse MCs or mouse testis was used to prepare complementary DNA (cDNA). PCR was done with oligonucleotide pairs for PIAS family members using 5 µl of cDNA. bp, base pairs. (B) 293T cells were cotransfected with 2 µg of plasmid expressing myc-E12 together with (+) or without (−), 2 µg of plasmid expressing HA-SUMO-1, and 2 µg of plasmid expressing GFP-PIAS1 (1), -PIAS3 (3), -PIASXα (Xα), -PIASXβ (Xβ) and -PIASy (y). Upper panel, Cell lysates were subjected to immunoblotting with anti-myc antibody. Middle and Lower panel, Cell lysates were immunoprecipitated (IP) with anti-myc antibody. The immunoprecipitates were subjected to SDS-PAGE and analyzed by Western blotting (WB) with anti-myc antibody. After ECL development, the filter shown in the middle panel was stripped and reproved with anti-HA antibody (lower panel). (TIF) [file pone.0041186.s001.tif]

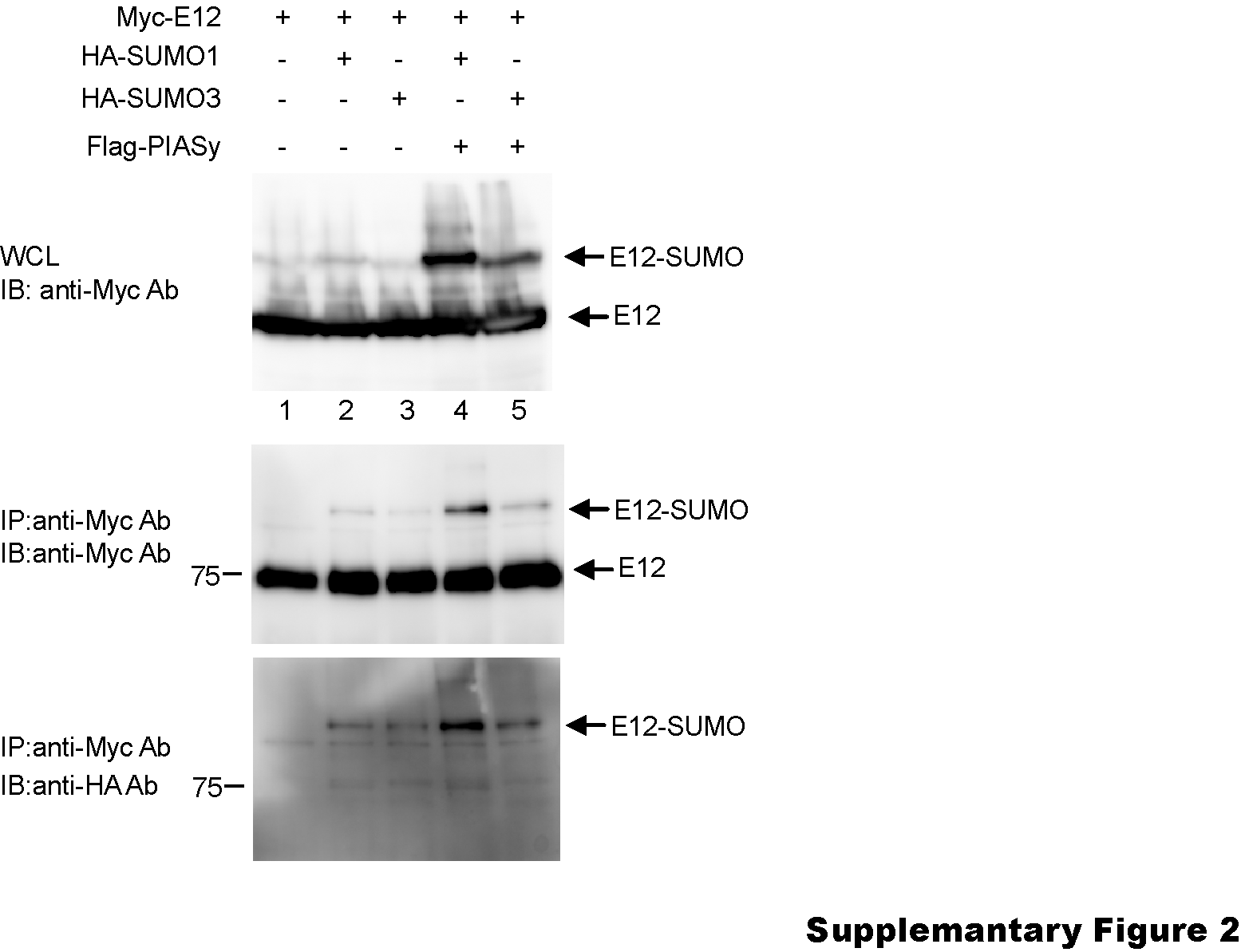

Supplement: Figure S2 — PIASy promotes SUMO-1 and SUMO-3 modification of E12 in vivo. 293T cells were cotransfected with (+) or without (−) 2 µg of plasmid expressing myc-E12, 2 µg of plasmid expressing HA-SUMO-1 or HA-SUMO-3, and 2 µg of plasmid expressing flag-PIASy. Upper panel, Cell lysates were subjected to immunoblotting with anti-myc antibody. Middle and Lower panel, Cell lysates were immunoprecipitated (IP) with anti-myc antibody. The immunoprecipitates were subjected to SDS-PAGE and analyzed by Western blotting (WB) with anti-myc antibody. After ECL development, the filter shown in the middle panel was stripped and reproved with anti-HA antibody (lower panel). (TIF) [file pone.0041186.s002.tif]
